# Quantifying conformational changes in GPCRs: glimpse of a common functional mechanism

**DOI:** 10.1186/s12859-015-0567-3

**Published:** 2015-04-23

**Authors:** James AR Dalton, Isaias Lans, Jesús Giraldo

**Affiliations:** Laboratory of Molecular Neuropharmacology and Bioinformatics, Institut de Neurociències and Unitat de Bioestadística, Universitat Autònoma de Barcelona, 08193 Bellaterra, Spain

**Keywords:** GPCR, Quantify, Conformational change, Receptor activation, Interhelical interaction, Dihedral angle

## Abstract

**Background:**

G-protein-coupled receptors (GPCRs) are important drug targets and a better understanding of their molecular mechanisms would be desirable. The crystallization rate of GPCRs has accelerated in recent years as techniques have become more sophisticated, particularly with respect to Class A GPCRs interacting with G-proteins. These developments have made it possible for a quantitative analysis of GPCR geometrical features and binding-site conformations, including a statistical comparison between Class A GPCRs in active (agonist-bound) and inactive (antagonist-bound) states.

**Results:**

Here we implement algorithms for the analysis of interhelical angles, distances, interactions and binding-site volumes in the transmembrane domains of 25 Class A GPCRs (7 active and 18 inactive). Two interhelical angles change in a statistically significant way between average inactive and active states: TM3-TM6 (by -9°) and TM6-TM7 (by +12°). A third interhelical angle: TM5-TM6 shows a trend, changing by -9°. In the transition from inactive to active states, average van der Waals interactions between TM3 and TM7 significantly increase as the average distance between them decreases by >2 Å. Average H-bonding between TM3 and TM6 decreases but is seemingly compensated by an increase in H-bonding between TM5 and TM6. In five Class A GPCRs, crystallized in both active and inactive states, increased H-bonding of agonists to TM6 and TM7, relative to antagonists, is observed. These protein-agonist interactions likely favour a change in the TM6-TM7 angle, which creates a narrowing in the binding pocket of activated receptors and an average ~200 Å^3^ reduction in volume.

**Conclusions:**

In terms of similar conformational changes and agonist binding pattern, Class A GPCRs appear to share a common mechanism of activation, which can be exploited in future drug development.

**Electronic supplementary material:**

The online version of this article (doi:10.1186/s12859-015-0567-3) contains supplementary material, which is available to authorized users.

## Background

G-protein-coupled receptors (GPCRs) are the largest superfamily of signalling molecules in the human genome with approximately 800 genes, each containing a seven-helix transmembrane domain (7TM) [1]. GPCRs are distributed throughout the body in cellular membranes and act as conduits, binding extracellular ligands (neurotransmitters, hormones, lipids, peptides, drugs) or capturing light photons (in the case of rhodopsin), converting the energy they embody into intracellular responses by stabilizing certain receptor conformational states, in turn influencing intracellular binding of guanine nucleotide-binding proteins (G-proteins) or β-arrestin [2,3]. As GPCRs are highly abundant in the human body and involved in many diverse signalling pathways (e.g. metabolic, regulatory, immunological, neurological), as well as typically binding small ligands, they serve as excellent drug targets [4]. GPCRs are currently targeted by ~40% of today’s marketed drugs and it is likely that their potential “druggability” is even greater [5]. Indeed, of the 370 non-olfactory GPCRs, 59 have already been drugged with small molecules [6].

Despite their known importance as drugs targets and role in human disease [5], the mechanisms that precisely control GPCR ligand binding and receptor activation have until very recently been hindered by a lack of structural knowledge, in particular due to a relative lack of crystallized active receptor states and receptor-ligand complexes. GPCRs are grouped into distinct classes with Class A (or rhodopsin-like) being the largest and containing the greatest number of crystallized structures, mostly in their inactive state. However, significant advances in crystallization has recently permitted the structural determination of several Class A receptors in active (agonist-bound and sometimes G-protein bound) states, i.e. β2-adrenergic [7], adenosine A2A [8], metarhodopsin II [9], neurotension NTS1 [10], acetyl choline muscarinic M2 [11], and P2Y12 [12] receptors. These active structures have significantly added diversity and detail to GPCR structural knowledge, which before them was primarily limited to the inactive structures of rhodopsin (first crystallized in 2000 [13]), antagonist-bound β2/1-adrenergic (crystallized in 2007 [14] and 2008 [15]) and adenosine A2A receptors (crystallized in 2008 [16]). Furthermore, in addition to the recent emergence of several active structures, several more Class A receptors have been crystallized in their inactive antagonist-bound state, e.g. mu- [17], kappa- [18], delta- [19], N/OFQ- [20] opioid receptors, muscarinic M2 receptor [21], chemokine receptors CXCR4 [22] and CCR5 [23], and protease-activated receptor 1 (PAR1) [24]. As a consequence, several studies have taken advantage of these recent developments in crystallization and attempted to identify various activation mechanisms in Class A GPCRs, primarily through visual comparison of specific receptor structures in inactive and active states [25-29]. Some studies have also identified specific “micro-switches”, i.e. small groups of residues that undergo conformational change during receptor activation [30-32]. Molecular dynamics has been used to investigate pathways of activation in Class A receptors, such as β2-adrenergic [33-35]), adenosine A2A [36], muscarinic acetylcholine M2 [37,38], S1P1 [39] and rhodopsin [40]. Several intrahelical features have also been identified as important in GPCR structure and function, such as kinks or α-bulges, often near prolines in highly conserved positions [41-43], and sulphur-containing residues acting as molecular gears [44]. Biased signalling, i.e. different ligands selecting for different intracellular binding partners, is another area that is gaining particular attention [45-48], such as β2-adrenergic receptor adopting different conformations of TM6 for selective Gi or Gs binding [49], enhancement of β-arrestin over G-protein binding with δ-opioid receptor through disruption of interhelical interfaces [19] and serotonin 2A receptor where hallucinogenic and non-hallucinogenic agonists respectively stabilize different conformations of an intracellular loop region implicated in partner binding [50].

**Figure 1:**
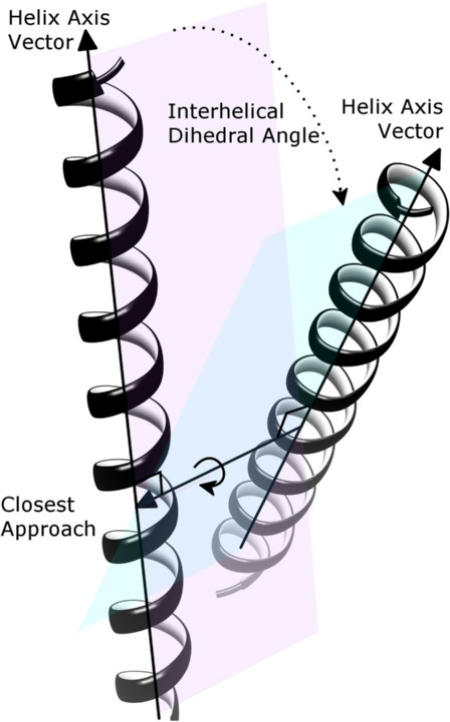
The interhelical dihedral angle: degree of rotation between planes of two interacting helices defined by axis vectors and perpendicular vector of closest approach. Helix pair interaction is assessed by number of interhelical van der Waals contacts and H-bonds.

However, although informative in their own way, none of these studies have quantitatively assessed GPCR conformational change at an overall level between active and inactive states, nor utilised the extra number of receptor structures that have recently become available to provide increased statistical power. Furthermore, in our opinion, an explanation of how the common functional mechanisms that appear to be at work in GPCR activation (e.g. suggested by [26]) are mediated through agonist (or reversed in antagonist) binding has not yet been convincingly made. Such an understanding would greatly aid the design of new or more selective/biased agonists or antagonists to target therapeutically important GPCRs, as well as explaining the effect that naturally occurring mutations have on constitutive receptor activity and disease, or indeed predicting where mutations could be inserted to intentionally modulate receptor activity. We believe the opportunity for such analyses has become possible due to the recent surge of new crystal structures in varying states of activation. Here, we present a quantifying analysis of GPCR conformational change with statistical comparison, using a set of 25 Class A structures: 7 active and 18 inactive and two algorithms: *Helix Packing Pair* [51] and POVME [52]. Our intention is to quantify existing theories of Class A GPCR activation, as well as potentially discovering new ones, and in doing so help to form a better understanding of the common features governing receptor activation. However, we do not attempt to replicate specific analyses of previous studies that focus on highly detailed aspects of particular receptors or nuanced differences. Instead we focus on overall conformational changes between TM helices, which can be identified as being in common across the whole Class A receptor family, as far as is currently possible with the structures available. We also seek to identify the most important aspects of conformational change according to statistical significance so that these can potentially be correlated with agonist or antagonist binding patterns. Regarding the algorithms we chose to employ, *Helix Packing Pair* mathematically measures the dihedral angle between packed helix pairs (see Figure 1 and Methods) as well as measuring the extent of atomic interaction and distances between helices [51], thereby detecting rotational and translational movements of each helix and their changing interactions. POVME measures binding pocket volume and shape and permits a mathematical comparison between different GPCR conformational binding-site volumes. As a result, by combining the various data, an internal geometrical picture of each GPCR can be made, which can then be compared to different conformations of a particular receptor or across different receptors (independently of structural superimposition, which can be prone to variation) or used to calculate average features across all known GPCR states. Here we specifically compare the geometrical features of active Class A GPCR structures with respect to inactive structures, both in terms of individual receptors and across the family as a whole, and as a result identify the most statistically significant and common conformational changes involved. Furthermore, with respect to five specific Class A receptors whose inactive and active states are both known, including the recently released P2Y purinoceptor 12 [12] and [53], we compare protein-agonist and protein-antagonist interactions and possibly identify a common means of agonist-mediated action that likely stabilizes the active state.

## Results

**Figure 2:**
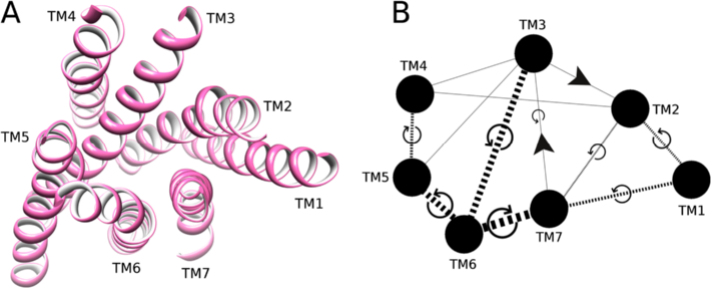
Helix pairs in Class A GPCRs. **(A)** Extracellular view of 7TM fold of the muscarinic acetylcholine M2 receptor (PDB *id:* 3UON). **(B)** 2D representation of average conformational changes during receptor activation (from 18 inactive and 7 active Class A GPCRs). Helices are solid black circles. Helix pairs connected with black dotted lines. Line width is proportional to change in interhelical angle. Circular arrows give angle rotation direction: anticlockwise for angle decrease (helices become more parallel), clockwise for angle increase (helices become less parallel). Solid black arrows show translational movement of helices.

**Figure 3:**
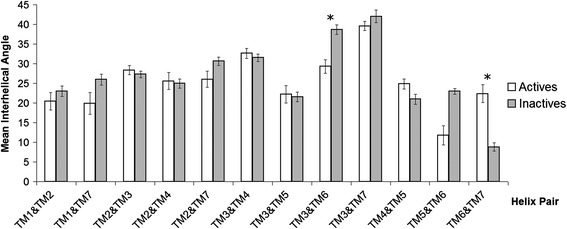
Average interhelical angles extracted from 18 inactive and 7 active class A GPCR structures. Standard error bars are shown. *denotes statistical significance between inactive and active states with Student’s *t*-test after Bonferroni correction for multiple testing.

**Figure 4:**
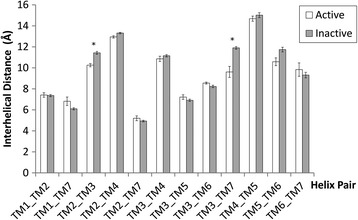
Average interhelical distances extracted from 18 inactive and 7 active class A GPCR structures. Distances are calculated between Cα atoms of central and highly conserved residues in the 7TM core: G/I1.46, D2.50, I/V3.40, W4.50, P5.50, F6.44, S/C7.46 (Ballesteros-Weinstein numbering [60]). Standard error bars are shown. *denotes statistical significance between inactive and active states with Student’s *t*-test after Bonferroni correction for multiple testing.

**Figure 5:**
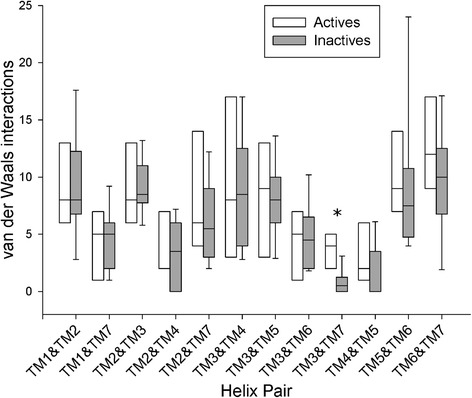
Boxplots of van der Waals atomic contacts between packed helix pairs in 7 active and 18 inactive Class A GPCR structures. *denotes statistical significance between inactive and active states with Wilcoxon rank-sum test after Bonferroni correction for multiple testing.

**Figure 6:**
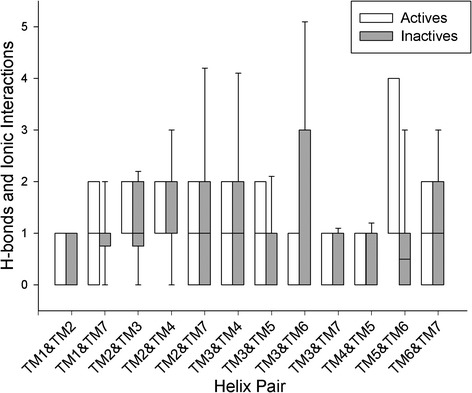
Boxplots of H-bonds and electrostatic contacts between packed helix pairs in 7 active and 18 inactive Class A GPCR structures.

In order to quantify conformational changes in 25 Class A GPCR crystal structures, 18 in the inactive state and 7 in the active state, an analysis was made with *Helix Packing Pair* [51] to determine the orientation of helix pairs in each 7TM domain and how these differ between receptor states. Three conformational features were assessed: (i) helix tilt by measuring degree of rotation between contacting helices, i.e. their interhelical angle from 0-90° (see Figure 1) [51], (ii) helix translation by measuring interhelical distances between centrally positioned and highly conserved residues in the core of each receptor (see Additional file 1: Figure S1I and Methods), (iii) interhelical interaction by counting the number of van der Waals’ (vdWs), hydrogen bonds and electrostatic contacts between helices. In total, in each receptor twelve helix pairs are commonly observed with TM3 participating in five pairs, TM2 and TM7 participating in four each, TM4, TM5 and TM6 in three each, and TM1 in two (see Figure 2). In this context, TM3 can be considered the central “hub” helix in the 7TM fold, making the most interhelical packing contacts. The interhelical angles, interhelical distances and interhelical contacts in twelve helix pairs were compared across all receptor structures with the average values for active and inactive receptor states shown in Figure 3 (angles), Figure 4 (distances), Figures 5 and 6 (vdWs and H-bonds, respectively). In addition, the differences between these states and hence overall conformational changes are shown proportionally in Figure 2. Overall the average interhelical angles involving TM3 remain mostly static across inactive and active states as its tilt and that of its interacting partners remain relatively constant. An exception to this rule is the TM3-TM6 angle, which undergoes a significant adjustment as the intracellular end of TM6 tilts outwards during receptor activation, becoming more parallel with respect to TM3 (an average angle of 40° ±1 s.e.m. for inactive states and an average of 31° ±2 s.e.m. for active states; *t*-test with Bonferroni correction for multiple comparisons: p = 0.002). However, TM3 undergoes a significant translational movement upwards and towards TM2, reducing the distance between residues L/I/V/T3.40 and D2.50 (an average distance of 11.4 Å ±0.1 s.e.m. for inactive states and an average of 10.3 Å ±0.2 s.e.m. for active states; *t*-test with Bonferroni correction: p = 0.001). The upward axial movement of TM3 has been proposed before as a component of receptor activation [26] but the sideways movement towards TM2 also appears to be an important feature, contributing to a more compact 7TM fold. As well as the interhelical angles involving TM3, the angles involving TM1, TM2 and TM4 remain mostly constant between active and inactive states, suggesting a degree of rigidity in this side of the receptor. However, noticeably greater differences are observed between active and inactive states with respect to TM5, TM6 and TM7. As already mentioned, the outward tilting of TM6 at the intracellular side seen in G-protein-bound activated receptors [7,9,11] (Additional file 1: Figure S2I) also creates key changes in interhelical angles: TM5-TM6 and TM6-TM7. The angle between TM5 and TM6 decreases as these helices become more parallel (an average of 24° ±1 s.e.m. in inactive receptors and an average of 15° ±3 s.e.m. in active receptors; *t*-test: p = 0.025, although not statistically significant after Bonferroni correction) while the angle between TM6 and TM7 increases as these helices become less parallel (an average of 9° ±1 s.e.m. in inactive receptors and an average of 21° ±2 s.e.m. in active receptors; *t*-test and Bonferroni correction: p = 0.0001). These angle differences also reflect a movement in the helices partnering TM6, i.e. TM5 undergoes a minor tilt outwards at its intracellular side and TM7 tilts inwards at its intracellular side in the reverse direction to TM6. The tilting of TM7 also causes a minor reduction in the TM1-TM7 angle as these two helices become more parallel (an average of 25° in inactive receptors compared to an average of 21° in active receptors; *t*-test p = 0.120). There is also a significant inward translational movement of TM7 towards TM3 (an average distance of 11.9 Å ±0.1 s.e.m. for inactive states and an average of 9.6 Å ±0.5 s.e.m. for active states; *t*-test with Bonferroni correction: p = 0.001), as well as a statistically significant increase in the number of vdWs between these two helices in active receptor states (Wilcoxon rank-sum test with Bonferroni correction for multiple comparisons: p = 0.009). Conformational change in TM7 can perhaps be considered the most defining features of GPCR activation as it is statistically significant across all three conformational categories and is seemingly independent of G-protein binding (only three of the seven active receptor states contain an intracellular binding partner, see Methods for details).

In general terms, the number of vdWs between packed helices is greater in active states relative to inactive states (Figure 5), suggesting a more compact fold in activated receptors. With regards to H-bonds the picture is more balanced with both losses and gains occurring across all helix pairs, although a loss of interaction between helices TM3 and TM6 is noticeable although not statistically significant (see Figure 6). This observation partly reflects the loss of the “ionic lock” that occurs in some receptors during activation as TM6 tilts outwards on the intracellular side away from TM3 [9,26]. Interestingly, the distance between TM3 and TM6 at their midpoints does not change between active and inactive states, suggesting that tilting of TM6 around its centre point is responsible for conformational change between TM3 and TM6 in G-protein-bound receptor structures (see Additional file 1: Figure S2I) rather than a translational movement. An increase in the number of H-bonds between TM5 and TM6 is also noticeable in active structures and is close to statistical significance (Wilcoxon rank-sum p-value = 0.097) as is a translational movement of TM5 towards TM6 (an average distance of 11.7 Å ±0.2 s.e.m. for inactive states and an average of 10.6 Å ±0.4 s.e.m. for active states; *t*-test p = 0.011, although not statistically significant after Bonferroni correction). These features are indicative of a stronger interaction between TM5 and TM6, which has previously been described in B2AR and M2R activation [25]. In general terms, it is striking how the distances between helix pairs at their centre points (see Additional file 1: Figure S1I) do not change much apart from the three examples mentioned: TM3-TM7, TM2-TM3 and TM5-TM6. Instead it appears that helix tilting is the primary means of imparting conformational change in Class A GPCRs, with changes in interhelical angles mainly involving TM5, TM6 and TM7.

**Figure 7:**
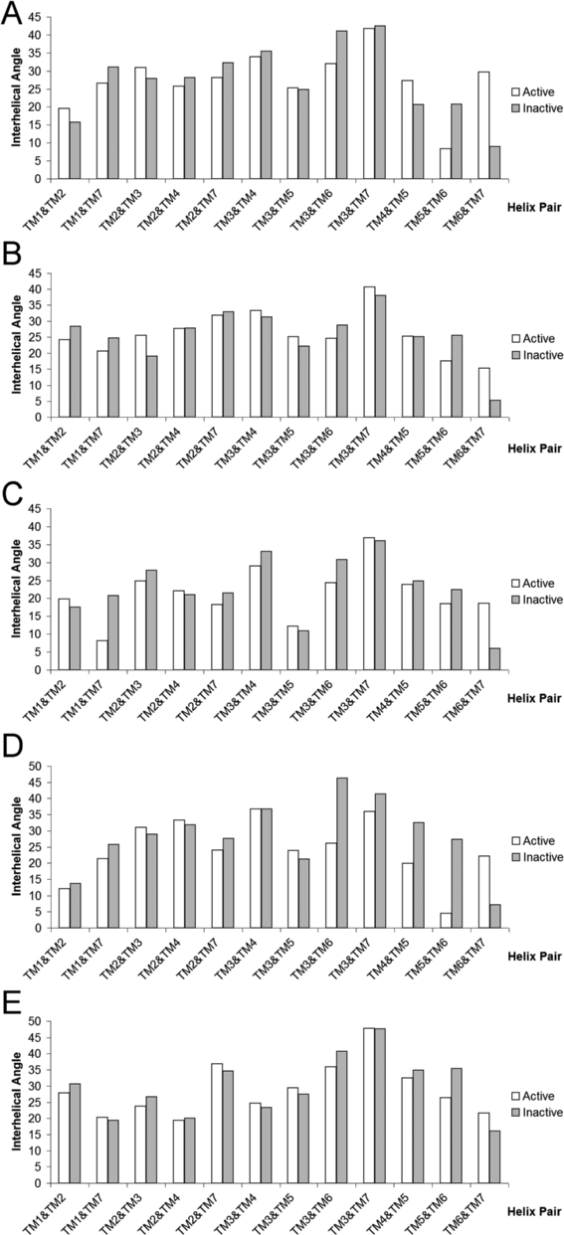
The difference between interhelical angles in inactive and active states of Class A GPCRs: **(A)** β2-adrenergic receptor, **(B)** rhodopsin, **(C)** adenosine A2A receptor, **(D)** muscarinic acetylcholine M2 receptor, **(E)** P2Y purinoceptor 12.

**Figure 8:**
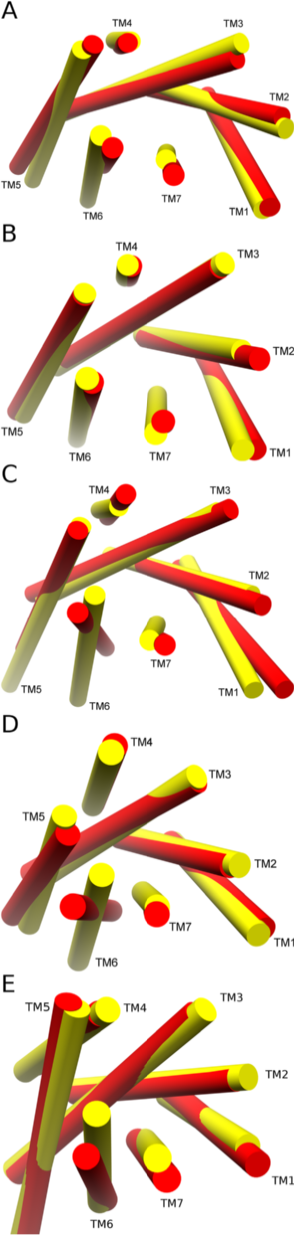
Comparison of 7TM conformation (from extracellular side, helices represented as cylinders calculated with CHIMERA [68]) in inactive (red) and active (yellow) states of Class A GPCRs: **(A)** rhodopsin, **(B)** adenosine A2A receptor, **(C)** β2-adrenergic receptor, **(D)** muscarinic acetylcholine M2 receptor, **(E)** P2Y purinoceptor 12.

In order to inspect conformational changes in more detail, interhelical angles and their effect on receptor conformation were assessed in the inactive and active states of five receptors: β2-adrenergic receptor (B2AR), adenosine A2A receptor (A2AR), muscarinic acetylcholine M2 receptor (M2R), Rhodopsin (RHO), and P2Y purinoceptor 12 (P2Y12R) (see Figures 7 and 8 for simplified representations of conformational change). These five receptors are the only GPCRs that have been crystallized in both active and inactive states, although A2AR and P2Y12R are only “semi-active” as they contain no bound G-protein or stabilizing nanobody. The interhelical angle changes observed in these five receptors reflect the averages as one might expect, although to differing degrees. The greatest conformational change is seen in the fully activated receptors with bound G-protein e.g. B2AR and M2R, and to a lesser extent RHO, which has a bound intracellular fragment. Some conformational change is also apparent in “semi-active” A2AR and P2Y12R, although it is more subtle. Taking each helix pair at a time, the greatest change between TM5 and TM6 occurs in M2R, where a shift of -22° is observed during activation (Figure 7D). The smallest change in the TM5-TM6 angle is observed in A2AR, where a shift of -4° is explained by a relative lack of movement in TM6 as a result of no bound G-protein (Figure 7C). Likewise, the greatest change in the TM3-TM6 angle occurs in M2R, with a shift of -20°, while the smallest change is seen in RHO with -4°. With regards to the TM6-TM7 angle, four receptors show a significant change upon agonist-induced activation, while in P2Y12R the change is more moderate. The greatest is observed in B2AR where TM6-TM7 shifts by +21° (Figure 7A) and is the lowest in P2Y12R with a shift of +4° (Figure 7E), a result of no bound G-protein and little intracellular movement of TM6. However, more unusually, inward bending of TM6 towards the orthosteric binding pocket on the extracellular side contributes to a change in the TM6-TM7 angle in P2Y12R. This demonstrates that although a common rotational change between TM6 and TM7 occurs in (semi-)activated receptors, this conformational movement can be delivered in different ways, either through a relative tilting of TM6 with respect to TM7 or vice versa, or indeed a combination of both in the larger conformational change seen in fully activated receptors, e.g. B2AR and M2R. Likewise, despite no bound G-protein, a significant change in the TM6-TM7 angle is observed in the “semi-active” state of A2AR with a shift of +13° (Figure 7C) due to an inward tilting of TM7 on the intracellular side. In particular, this suggests that an intracellular movement of TM7 is a pre-requisite step for full activation, which is followed by intracellular movement of TM6 and G-protein binding. All five receptors show an increased level of interaction between TM3 and TM7, in agreement with average observations, with the greatest change seen in B2AR and M2R, reflecting their fully active status (see Additional file 1: Figure S3I). Likewise, the interhelical TM3-TM7 distance decreases in all receptors with the greatest change seen in A2AR, where residue S7.46 undergoes a shift towards I3.40 by 5.3 Å (Additional file 1: Figure S4I). However, in other receptors a shift of 1.5-2.4 Å is more common.

**Figure 9:**
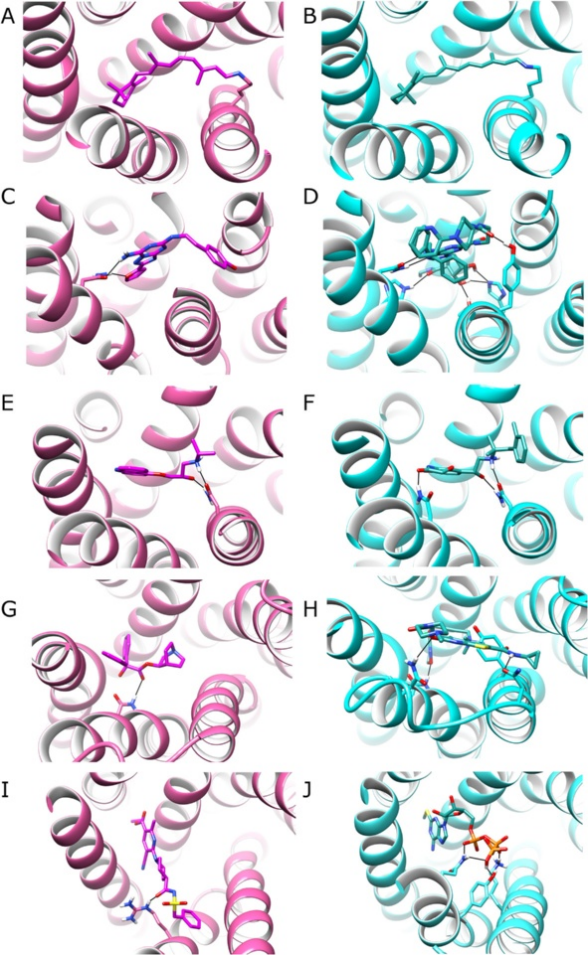
A comparison of antagonists (magenta) in inactive states (pink) and agonists (teal) in active states (cyan) of Class A GPCRs: **(A, B)** rhodopsin, **(C, D)** adenosine A2A, **(E, F)** β2-adrenergic, **(G, H)** muscarinic acetylcholine M2, **(I, J)** P2Y purinoceptor 12. Only side-chains on TM6 and TM7 that make covalent or H-bonds with antagonists/agonists are displayed. H-bonds shown as black lines, calculated with CHIMERA [68]. Agonists typically make more H-bonds with TM6/7 than respective antagonists.

In view of the observed movements of TM7 in relation to TM3 and TM6, the interaction of TM6 and TM7 with co-crystallized agonists and antagonists was compared in five receptors (see Figures 8 and 9). RHO is different from the others as its ligand (retinal) is covalently bound to TM7 and undergoes a conformational switch upon photo-activation, contributing to a different orientation of TM7 (Figures 8A and 9A-B). The four other receptors bind diffusible agonists/antagonists and changes in the orientation of TM7 are observed between inactive and active states in all (Figure 8). In the active state of A2AR, where TM7 makes an intracellular inward tilt with respect to TM6 (Figure 8B), the agonist UK-432097 makes three H-bonds with TM7, whilst in the inactive state, the antagonist ZM241385 makes none (Figure 9C-D). In addition, the agonist also makes an extra H-bond with TM6. In the active and inactive states of B2AR, the agonist (BI167107) and antagonist (carazolol) both make two H-bonds with TM7, however unlike the antagonist, the agonist makes an H-bond with TM6 (Figure 9E-F). This may contribute extra stability to the different orientation between TM6 and TM7 (Figure 8C). Furthermore, the agonist in B2AR is shorter in length than its respective antagonist, requiring a translation of TM7 to maintain the H-bonding pattern. In the active state of P2Y12R, an inward movement of TM7 is concomitant with the agonist (2-methylthio-adenosine-5′-triphosphate) making an H-bond with TM7, while an extracellular inward movement of TM6 accommodates three H-bonds between agonist and TM6 (Figures 8E and 9J). These interactions are absent in the inactive state of P2Y12R, where the antagonist (AZD1283) makes only a single H-bond with TM6 (Figure 9I). Finally, M2R is different from the other receptors as the crystal structure of the active state contains an allosteric modulator (LY2119620) as well as an agonist (iperoxo) (Figure 9H). Both the antagonist (3-quinuclidinyl-benzilate) and iperoxo make a classical H-bond with TM6 (Figure 9G-H) while the latter also forms a close CH--O interaction with Y7.39 (on TM7). Furthermore, LY2119620 makes two H-bonds with TM6 and TM7, respectively (Figure 9H). These additional allosteric interactions may stabilise the observed change in the TM6-TM7 angle (Figure 8D). Therefore, taken together, the observed pattern of H-bonding interactions in these five receptors suggests agonist interactions with TM6 and TM7 encourage a shift in the TM6-TM7 interhelical angle, while H-bonds with TM7 additionally encourage a reduction in the TM3-TM7 distance, both seemingly common features of receptor activation, likely constituting the principal mechanism of agonist action.

**Figure 10:**
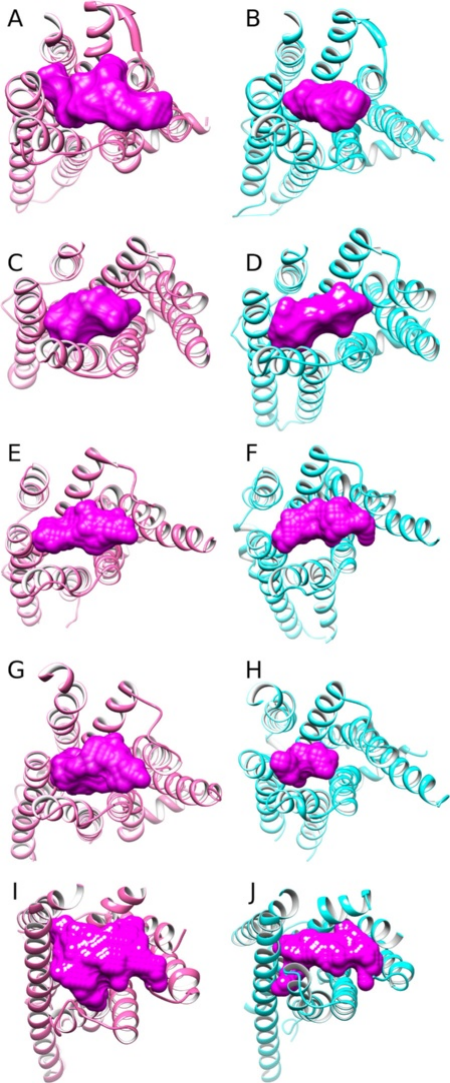
A comparison of inactive states (in pink) and active states (in cyan) of Class A GPCRs (extracellular view): **(A)** and **(B)**: adenosine A2A receptor, **(C)** and **(D)**: rhodopsin, **(E)** and **(F)**: β2-adrenergic receptor, investigate pathways of activation**(G)** and **(H)**: muscarinic acetylcholine M2 receptor, **(I)** and **(J)**: P2Y purinoceptor12. Binding-site volumes (in magenta) were calculated with POVME [52].

**Figure 11:**
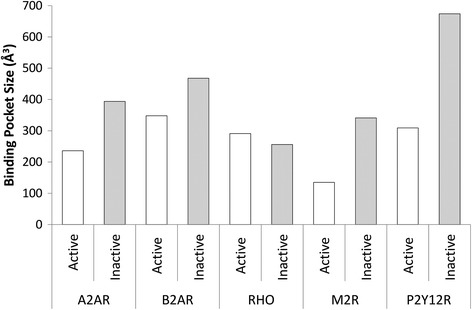
Binding pocket volumes (Å^3^) in the inactive and active states of Class A GPCRs: β2-adrenergic receptor (B2AR), rhodopsin (RHO), adenosine A2A receptor (A2AR), muscarinic acetylcholine M2 receptor (M2R), P2Y purinoceptor 12 (P2Y12R).

In order to investigate the effect changes in the TM3-TM6, TM5-TM6 and TM6-TM7 interhelical angles, as well as TM3-TM7 distance, have on the orthosteric binding-site, pocket volume in five receptors: B2AR, A2AR, M2R, RHO, and P2Y12R was calculated with POVME [52] in inactive and active states, respectively. This reveals a common conformational event that occurs during activation in terms of both pocket shape and volume (Figures 10 and 11). Regarding shape, the binding-site in each receptor narrows as a result of the inward transition of TM7. Also in four of five receptors, pocket volume significantly decreases during activation by an average of -212 Å^3^ ± 108 s.e.m. (*t*-test p = 0.029), a consequence of angle changes involving TM5, TM6 and TM7 and translation of TM7 and TM3. The one exception to this rule is rhodopsin whose pocket volume remains constant despite undergoing conformational narrowing (Figure 10A-B). Rhodopsin may be a special case as unlike the other receptors it does not bind a diffusible ligand and has an internalized extracellular loop 2, which traverses inside its 7TM domain. As a result, rhodopsin has the smallest pocket compared to other receptors (38-75% the size). This unusual feature likely restricts rhodopsin from making more extensive conformational changes upon receptor (in)activation and without needing to (un)bind its ligand, perhaps removes the need for it to do so. The significant conformational shift of TM7 is seemingly mediated by increased interactions with agonists, which results in an increase in the TM6-TM7 angle, reduction in TM3-TM7 distance and a narrower pocket. The extent of pocket shrinkage during activation varies between receptors, observed at its greatest in M2R whose pocket decreases by -60%, followed by P2Y12R whose pocket decreases by -54%, -40% in A2AR and -26% in B2AR (Figure 11). Nevertheless despite these differences in magnitude, it appears that the five receptors function in a similar fashion, both in terms of their relative reorientation of TM7, the binding mode of the agonist, and overall effect on binding-pocket shape and volume. These suggest a common functional mechanism.

The intracellular G-protein binding-site of Class A GPCRs generally gets less attention than the orthosteric ligand binding-site, although recent studies are addressing this [49,50,54]. In recognition, we also focussed POVME on the intracellular side of the five receptors in order to inspect conformational changes in the G-protein binding-site. As three receptors are co-crystallized with a bound intracellular partner (B2AR, M2R and rhodopsin), we expected to see strong intracellular changes and reassuringly we did (see Additional file 1: Figures S5I and S6I). In these three receptors, the intracellular G-protein binding-site increases in volume by an average of 565 Å^3^ ± 20 s.e.m. (*t*-test p = 0.001) from inactive to active state. This number is interesting as it is approximately twice that of the observed average decrease in the orthosteric binding pocket upon agonist binding. This supports the notion that conformational changes are amplified or “doubled” from extracellular to intracellular sides [55]. A2AR and P2Y12R, who have no co-crystallized intracellular partner, and despite apparent conformational changes on their extracellular sides, show no statistical volume changes in their intracellular G-protein binding-sites. However despite this, there is clear intracellular conformational change, particularly with respect to A2AR, whose outward tilting of TM6 and inward pinching of TM7 creates a flatter, wider space (see Additional file 1: Figure S5I). Binding of a G-protein would therefore likely stabilize an even greater displacement of TM6 and create a larger volume difference. P2Y12R also shows some intracellular conformational differences but these are a result of subtle changes in the C-terminal structure of TM7 rather than helix tilting, with a partial unravelling of TM7 by half a helix-turn also changing the orientation of H8. This has an effect of increasing the space between TM7 and TM2 and changing the shape of the pocket but not its overall volume (Additional file 1: Figure S5I). However, in way of caution, both the inactive and active states of P2Y12R are crystallised with BRIL-fusion constructs (between TM5 and TM6), meaning intracellular conformational changes may be restricted.

## Discussion and conclusions

Although comparative studies of GPCRs in active and inactive states have been performed before [25-29], these have generally been based on visual comparisons of structural superimpositions, which can lead to different interpretations. This is particularly the case when conformational changes in receptors are large or comparison between different receptors is difficult because of alternative structural alignments. Here, we implement quantifying methods for GPCR conformational analysis: *Helix Packing Pair* [51] and POVME [52] that operate independently of structural superimposition as each receptor structure is analysed internally, making for easier comparison between active and inactive states. It also allows for a statistical evaluation of the average differences between receptor states.

Despite the calculations involving different receptors, as Class A GPCRs share many sequence and structural features as well as G-protein binding partners [56], we believe such comparisons are meaningful and can potentially reveal common conformational changes and shared mechanisms of agonist/antagonist action. The results generated with a set of 25 Class A GPCRs, and subset of five receptors in detail, suggest a common overall set of conformational changes that occur during GPCR activation, and that these are mediated by agonist interaction with TM6 and particularly TM7. The statistically significant conformational changes in active states, with respect to inactive, can be summarized as follows: (i) an inward translation of TM7 with respect to TM3 and tilting of TM7 with respect to TM6, increasing the TM6-TM7 angle and increasing the interaction between TM3 and TM7; (ii) a lateral and upward translation of TM3, decreasing the distance between TM2 and TM3; (iii) stabilized by G-protein binding, an outward tilting of TM6 that increases the TM6-TM7 angle and decreases the TM3-TM6 angle (this also has trending effects of decreasing the TM5-TM6 angle, decreasing TM3-TM6 interaction and increasing TM5-TM6 interaction). In an analysis with POVME in both active and inactive states, the sum of these conformational changes results in the narrowing of the orthosteric binding-site between TM3 and TM6/TM7, significantly decreasing pocket volume. On the intracellular side, significant increases in volume of the G-protein binding-site are observed in activated receptors with bound G-protein or derivatives, which are approximately twice the size in magnitude of changes on the extracellular side. Even in receptors with no bound G-protein, some intracellular conformational change is apparent, which may assist G-protein binding. These observed changes suggest common conformational change is apparent in an overall sense of Class A GPCRs, which is mirrored by a repeating trend of increased H-bonding by agonists to TM6 and TM7 with respect to antagonists. Indeed, it is tempting to speculate that design of agonists through rational means should include functional H-bonding groups for strong contact with residues at the C-terminus of TM6 and the N-terminus of TM7. Likewise, antagonists should possibly be designed to preclude H-bonding with the N-terminus of TM7 and to a lesser extent with TM6 as well, although potentially still H-bonding with the N-terminus of TM3, which may act as an “anchor”.

**Figure 12:**
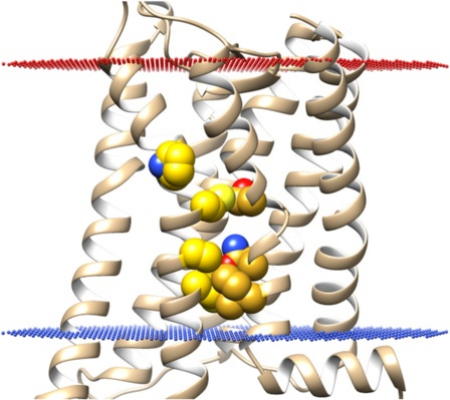
TM6-TM7 interface of Rhodopsin in its inactive state (PDB id: 1GZM) with residue positions 6.40, 6.43, 6.47, 6.50, 7.45, 7.49, 7.52 highlighted (in yellow/gold) (Ballesteros-Weinstein numbering [60]). Upon mutation these “hotspot” residues increase constitutive activity of some Class A GPCRs by destabilising their inactive states. Extracellular side of membrane is represented by red dots and the intracellular side by blue dots.

Another interesting aspect of GPCRs is the contribution this family of receptors makes to human disease through naturally occurring mutations that result in loss or gain of constitutive activity. Strikingly, of those mutations that are documented in various Class A GPCRs, a large proportion are located on TM6 and TM7, particularly at their mutual interface, which appears to confirm the functional importance of TM6 and TM7 conformational change. Other mutations are also found on TM3 or TM5 in their respective interfaces with TM6 or TM7 [57-59]. Some mutations directly affect ligand binding, for example, mutation of K7.43 or E3.49 (Ballesteros-Weinstein numbering [60]) in rhodopsin affects retinal binding and eradicates the salt bridge formed between TM3 and TM7 that stabilizes the inactive state [59]. Other residues are found on the intracellular side of TM6, such as D6.30, which forms the ionic lock between TM3 and TM6 e.g. in follicle-stimulating hormone receptor (FSHR), thyrotropin receptor (TSHR) and N6.30 in melanocortin 4 receptor (MC4R), whose mutations cause destabilization of the inactive state [59]. However, the majority of residues are located midway on helices TM6 and TM7 at their common interface e.g. M/L6.40 (rhodopsin, TSHR, MC4R), T/V6.43 (Luteinizing hormone receptor or LHR, TSHR, MC4R), C6.47 (LHR), P6.50 (TSHR), N7.45, N7.49, and L7.52 (TSHR) [58,59]. Mutation of any of these residues results in increased constitutive receptor activity by disrupting the packing between TM6 and TM7 and possibly by interfering with a water-mediated interhelical H-bonding network in inactive receptor states e.g. TSHR and rhodopsin [61,62]. It can be further speculated that mutation(s) in this “hotspot” area (see Figure 12) would likely affect activity of other Class A GPCRs. Unfortunately, studying the exact nature of the water-mediated effect between TM6 and TM7 in a statistical way is not possible as the crystal structure resolution of the majority of receptors is not high enough (2.2-3.5 Å). However the high-resolution crystal structures of inactive A2AR(-BRIL fusion) and inactive delta opioid receptor [19] have recently been determined (both at 1.8 Å resolution) [63] and show three water molecules bound between N7.45, N7.49 and L6.43, and one water molecule next to P6.50 (see Additional file 1: Figure S7I). It can be speculated that these water molecules exist in other Class A GPCRs stabilising their inactive states, and need to be rearranged for receptor activation when the TM6-TM7 interhelical angle changes or when residues in this interface are mutated. Indeed, molecular dynamics studies of water molecules in Class A GPCRs have recently shown that a continuous central water channel opens up during receptor activation, which is not present in the inactive state [64-67]. This highlights the functional role of dynamic waters in receptor activation although a high-resolution crystal structure of a Class A GPCR in its active state is ideally required to confirm this hypothesis. In addition to the TM6-TM7 interface, mutations in other interfaces alter the activity of some Class A GPCRs. In particular, mutations are often found in the TM3-TM6, TM5-TM6 and TM1-TM7 interfaces whose respective interhelical angles are seen to follow trends in receptor activation e.g. A6.34 in the interface with TM5 (LHR and TSHR) whose mutation causes a gain in receptor activity and Y6.52 (GNRHR, gonadotropin-releasing hormone receptor) whose mutation causes a loss of activity. Similarly located on TM6 in the interface with TM3 is D6.44 (LHR, TSHR) whose mutation causes a gain in receptor activity [58,59]. Conversely, on TM5 in the interface with TM6 is F5.48 (MC4R), whose mutation results in a loss of receptor activity [59]. Finally, located on TM7 and involved in the interface with TM1 is C7.47 (TSHR) whose mutation causes a gain in activity [58]. Taken together, these mutations point to the functional importance of several interhelical conformational changes, primarily involving interhelical angles TM6-TM7 and TM3-TM6 but also TM5-TM6 and TM1-TM7.

The analysis performed here also potentially offers a new means of classifying GPCR structural states. For instance, in active (and semi-active) receptors, the TM6-TM7 angle resides between 15-30°, TM5-TM6 between 8-21° and TM3-TM6 between 24-35°, while the TM2-TM3 and TM3-TM7 distances reside between 9.8-10.5 Å and 7.5-10.9 Å, respectively. On the other hand, in inactive receptors the TM6-TM7 angle adopts 0-19°, TM5-TM6 between 19-35° and TM3-TM6 between 31-46°, while TM2-TM3 and TM3-TM7 distances reside between 10.4-12.7 Å and 10.8-12.9 Å, respectively. Although these ranges overlap to some degree, as more active GPCR crystal structures become available these features can likely be refined further, as well as the statistical analysis between receptor states made even more robust. However, they currently offer a useful guide for classifying conformational states and extent of (in)activation of GPCR structures through quantifiable means without the need for visual comparison of structural superimpositions.

The recently developed strategy of crystallizing GPCRs in active states with co-crystallized G-proteins/nanobodies has made it possible for quantifying Class A GPCR activation with statistical significances. This has been aided by the recent crystallization of agonist-bound receptor structures that can be classified as “semi-active”. These structures show conformational changes on the extracellular side just like fully active structures but less noticeably on the intracellular side. They are particularly interesting as they give a suggestion of the intermediate steps that occur during receptor activation prior to G-protein binding. Clearly as more GPCRs are crystallized in their active state, statistical analysis of GPCR activation will become more powerful and potentially reveal more of the mechanistic subtleties involved, as well as identifying other common or differential aspects between receptors. However, it seems apparent that in terms of overall conformation change, Class A GPCRs share several features, as well as similar agonist/antagonist binding patterns, which can possibly be used as a starting point for the design of new drugs with predictable action against other Class A GPCRs.

## Methods

A non-redundant Class A GPCR dataset (where each receptor is only represented once in either active or inactive state, or twice in both active and inactive states) was constructed from all available PDB structures. The dataset consists of 18 Class A GPCR structures in their inactive state: rhodopsin (PDB *id:* 1GZM), adenosine A2A receptor (PDB *id:* 3PWH), β2-adrenergic receptor (PDB *id:* 2RH1), β1-adrenergic receptor (PDB *id:* 2VT4), squid rhodopsin (PDB *id:* 2Z73), histamine H1 receptor (PDB *id:* 3RZE), sphingosine1-phosphate receptor 1 (PDB *id:* 3V2Y), dopamine D3 receptor (PDB *id:* 3PBL), CXCR4 chemokine receptor (PDB *id:* 3ODU), M2 muscarinic acetylcholine receptor (PDB *id:* 3UON), M3 muscarinic acetylcholine Receptor (PDB *id:* 4DAJ), protease-activated receptor 1 (PDB *id:* 3VW7), kappa opioid receptor (PDB *id:* 4DJH), mu-opioid receptor (PDB *id:* 4DKL), nociceptin/orphanin FQ opioid receptor (PDB *id:* 4EA3), delta opioid receptor (PDB *id:* 4N6H), CCR5 chemokine receptor (PDB *id:* 4MBS), P2Y12 receptor (PDB *id:* 4NTJ); and 7 Class A GPCR structures in their active state: β2-adrenergic receptor (PDB *id:* 4LDE), metarhodopsin II (PDB *id:* 3PQR), adenosine A2A receptor (PDB *id:* 3QAK), neurotensin NTS1 receptor (PDB *id:* 4GRV), M2 muscarinic acetylcholine receptor (PDB *id:* 4MQT), serotonin 5-HT2B receptor (PDB *id:* 4IB4), P2Y12 receptor (PDB *id:* 4PY0). β2-adrenergic receptor, metarhodopsin II and M2 muscarinic acetylcholine receptor contain a co-crystallised intracellular binding partner (G-protein or fragment) and are therefore fully active. The other four receptors in the active category are considered “semi-active”. Criteria for selecting specific receptor structures was based on a multi-factorial assessment of: crystal structure resolution (lowest preferable), structural completeness, presence of co-crystallised agonist or antagonist, minimal unnatural amino acids or artificial mutations, absence of fusion constructs where possible e.g. lysozyme or BRIL, which are typically fused between TM5 and TM6 to aid crystallization. In rare instances where electron density is missing for particular residues, side-chains were completed with CHIMERA [68] by identifying the most probable rotamer from the Dunbrack backbone-dependent rotamer library [69]. The average X-ray crystallography resolution for active GPCR structures is 2.92 Å (S.D. ± 0.27 Å) and for inactive structures 2.77 Å (S.D. ± 0.40 Å). These values were considered acceptable for statistical comparisons to be made between receptor states.

The software *Helix Packing Pair* [51] was used to calculate the “global” interhelical angle between common packed helix pairs i.e. helices that contain at least one vdW interaction between helices. Helices were defined by DSSP [70] and water and lipids were removed. The interhelical angle is defined as the dihedral angle between two planes, where each plane is defined by the helix axis vector and the vector of closest approach, with the latter calculated as perpendicular to both helix axes (see Figure 1). Helix axis vectors are calculated by averaging all “local” axis vectors, each of which is calculated every four consecutive Cα atoms in a helix. Therefore local helix distortions and bends are reflected in the overall helix axis vector. The extent of interaction between helices is assessed by number of van der Waals (within 106% of the sum of vdW radii), H-bonds and ionic interactions (within 106% of distances: 2.70 Å for O···H-O bonds, 2.88 Å for O···H-N, and 3.10 Å for N···H-N). Interhelical distances were calculated between the Cα atoms of centrally-positioned (and highly conserved) residues in each helix. These coordinates constitute a plane through the middle of the 7TM core consisting of residues: 1.46, 2.50, 3.40, 4.50, 5.50, 6.44, 7.46 (Ballesteros-Weinstein numbering [60]) (see Additional file 1: Supporting Data). POVME 2.0 [52] was used to calculate binding-pocket volumes both on the extracellular and intracellular sides with default parameters. The intracellular binding-pocket was defined as the space between Intracellular Loop 2, C-termini of TM3, TM5 and TM7, and N-termini of TM2 and TM6. The extracellular binding-pocket was defined as the space beneath Extracellular Loop 2 and between N-termini of TM3, TM5 and TM7, and C-termini of TM2 and TM6. Protein-ligand H-bond analysis was made with CHIMERA [68] using default parameters, which utilise geometric criteria extracted from a crystal survey [71] for H-bond detection and additional “relax constraints” of 0.4 Å and 20° for tolerance.

### Statistical analyses

The variation of interhelical angles between helix pairs was assessed with mean values and standard errors, with statistical significance between active and inactive states measured with Student’s t-tests. The variation of the number of interhelical interactions (vdWs, H-bonds, ionic) was assessed with median values and interquartile ranges, with statistical significance between active and inactive states measured with Wilcoxon rank-sum tests because the non-continuous nature of the variables involved requires a nonparametric framework. For both parametric and nonparametric tests the Bonferroni correction for multiple comparisons was applied. Statistical significance was set at p < 0.05. Graphs were constructed with SigmaPlot 11.2 (Systat Software Inc., San Jose, CA U.S.A.) and statistical analyses were performed with SAS statistical package (SAS/STAT® 9.2, SAS Institute Inc., Cary, NC, U.S.A.).

## Additional file

Additional file 1:
**Contains supporting graphics of central core residues of rhodopsin, G-protein bound to β2-adrenergic receptor, interhelical atomic contacts and distances, intracellular G-protein binding-site volumes, water-mediated H-bonding network in Adenosine A2A Receptor, and an ensemble of active and inactive receptor conformations.**
