# Supplementary material for: Quantifying conformational changes in GPCRs: glimpse of a common functional mechanism
Source: BMC Bioinformatics. 2015 Apr 23;16(1):124. doi: 10.1186/s12859-015-0567-3 (PMC4422131; doi:10.1186/s12859-015-0567-3)
Supplement: Additional file 1: — Contains supporting graphics of central core residues of rhodopsin, G-protein bound to β2-adrenergic receptor, interhelical atomic contacts and distances, intracellular G-protein binding-site volumes, water-mediated H-bonding network in Adenosine A2A Receptor, and an ensemble of active and inactive receptor conformations. [file 12859_2015_567_MOESM1_ESM.pdf]

Quantifying Conformational Changes In GPCRs: Glimpse Of A Common Functional Mechanism

*James A.R. Dalton, Isaias Lans, Jesús Giraldo**

Laboratory of Molecular Neuropharmacology and Bioinformatics, Institut de Neurociències and Unitat de Bioestadística, Universitat Autònoma de Barcelona, 08193 Bellaterra, Spain.

* corresponding author

E-mail: Jesus.Giraldo@uab.es

SUPPORTING DATA

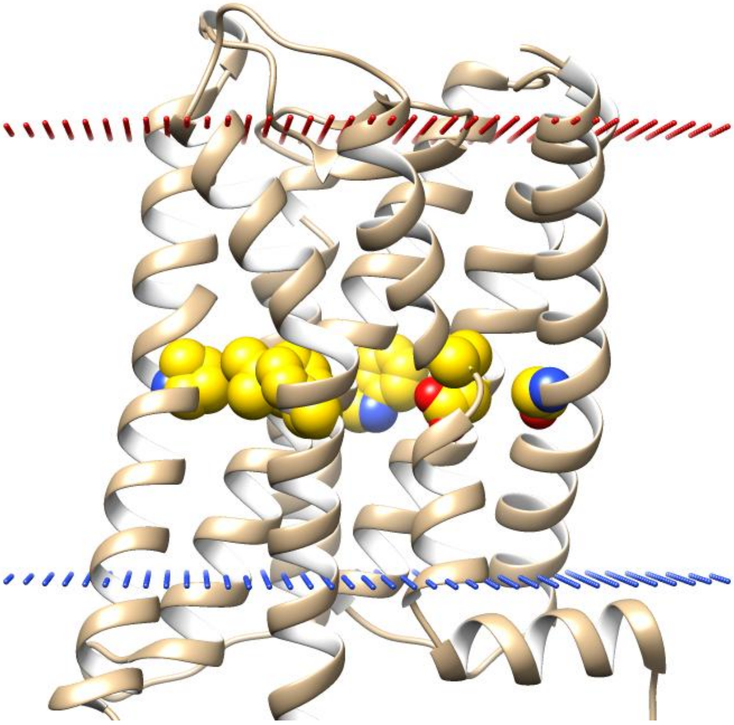

SI Fig. 1 Visualisation of the central core of Rhodopsin defined by residue positions 1.46, 2.50, 3.40, 4.50, 5.50, 6.44, 7.46 (in gold) (Ballesteros-Weinstein numbering [1]). The extracellular side of the membrane is represented as red dots and the intracellular side by blue dots.

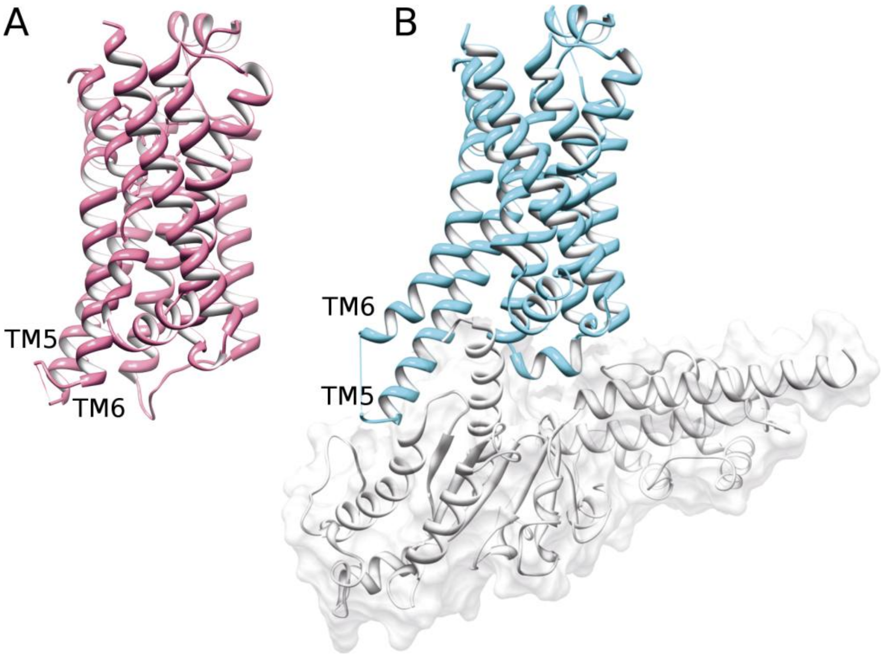

SI Fig. 2 A comparison of the inactive (in pink) and active (in cyan) states of the ß2-adrenergic receptor (transmembrane side-view, PDB id: 3SN6). The bound G-protein in the active state is shown in grey. TM5 and TM6 helices are labeled.

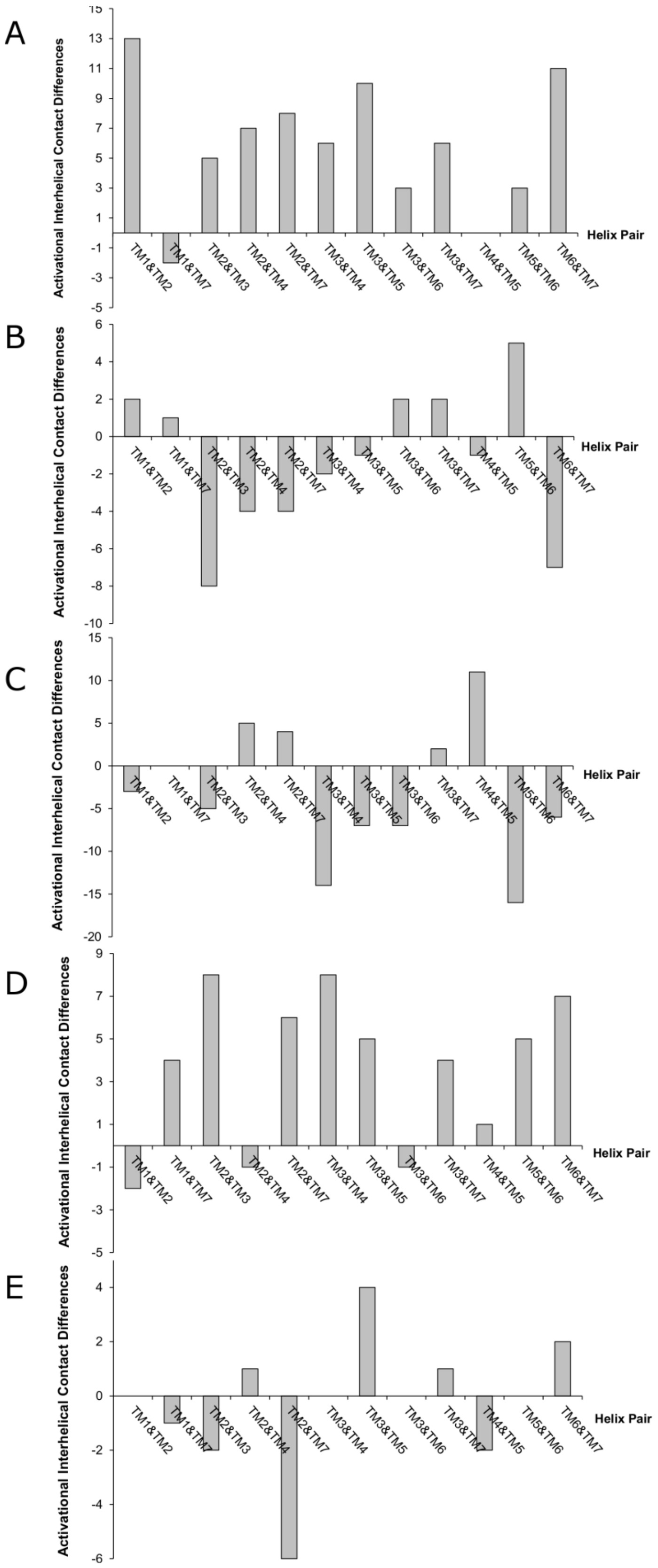

SI Fig. 3 Differences in interhelical atomic contacts (vdWs, H-bonds, electrostatic) in the inactive and active states of Class A GPCRs: (A) ß2-adrenergic receptor, (B) rhodopsin, (C) adenosine A2A receptor, (D) muscarinic acetylcholine M2 receptor, and (E) P2Y purinoceptor 12.

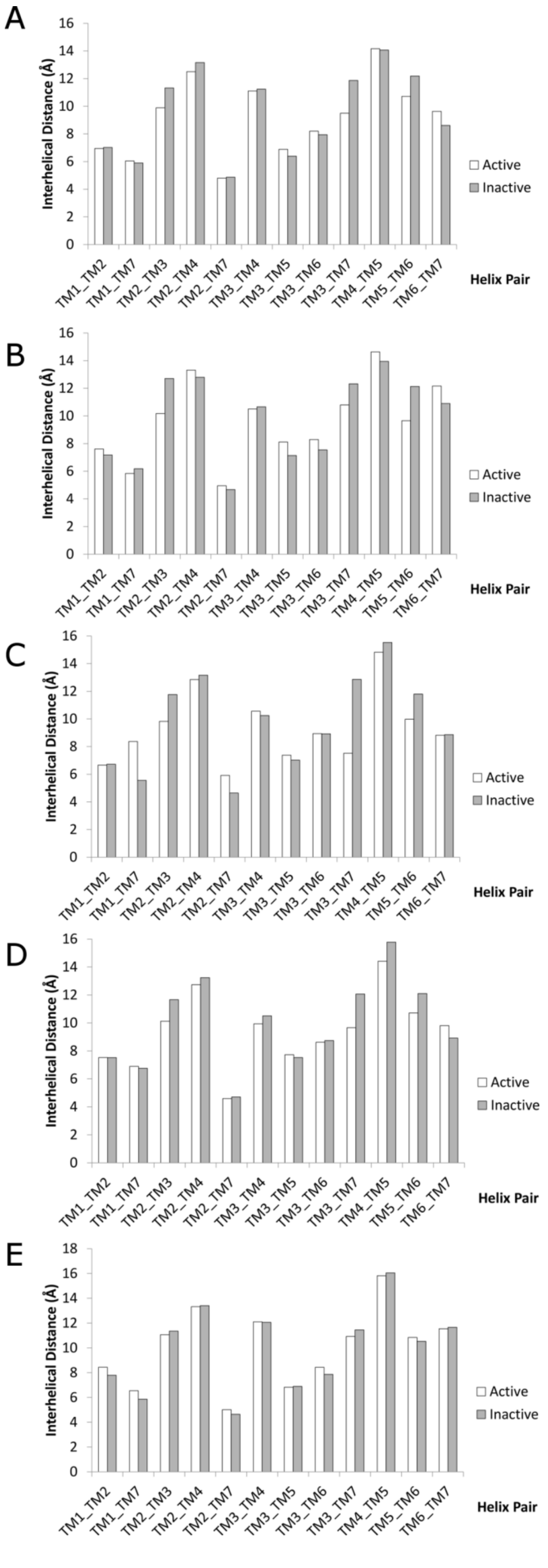

SI Fig. 4 Average interhelical distances in inactive and active states of Class A GPCRs: (A) ß2-adrenergic receptor, (B) rhodopsin, (C) adenosine A2A receptor, (D) muscarinic acetylcholine M2 receptor, (E) P2Y purinoceptor 12. Distances calculated between Cα atoms of (semi-)conserved residues in the planar core of the 7TM fold.

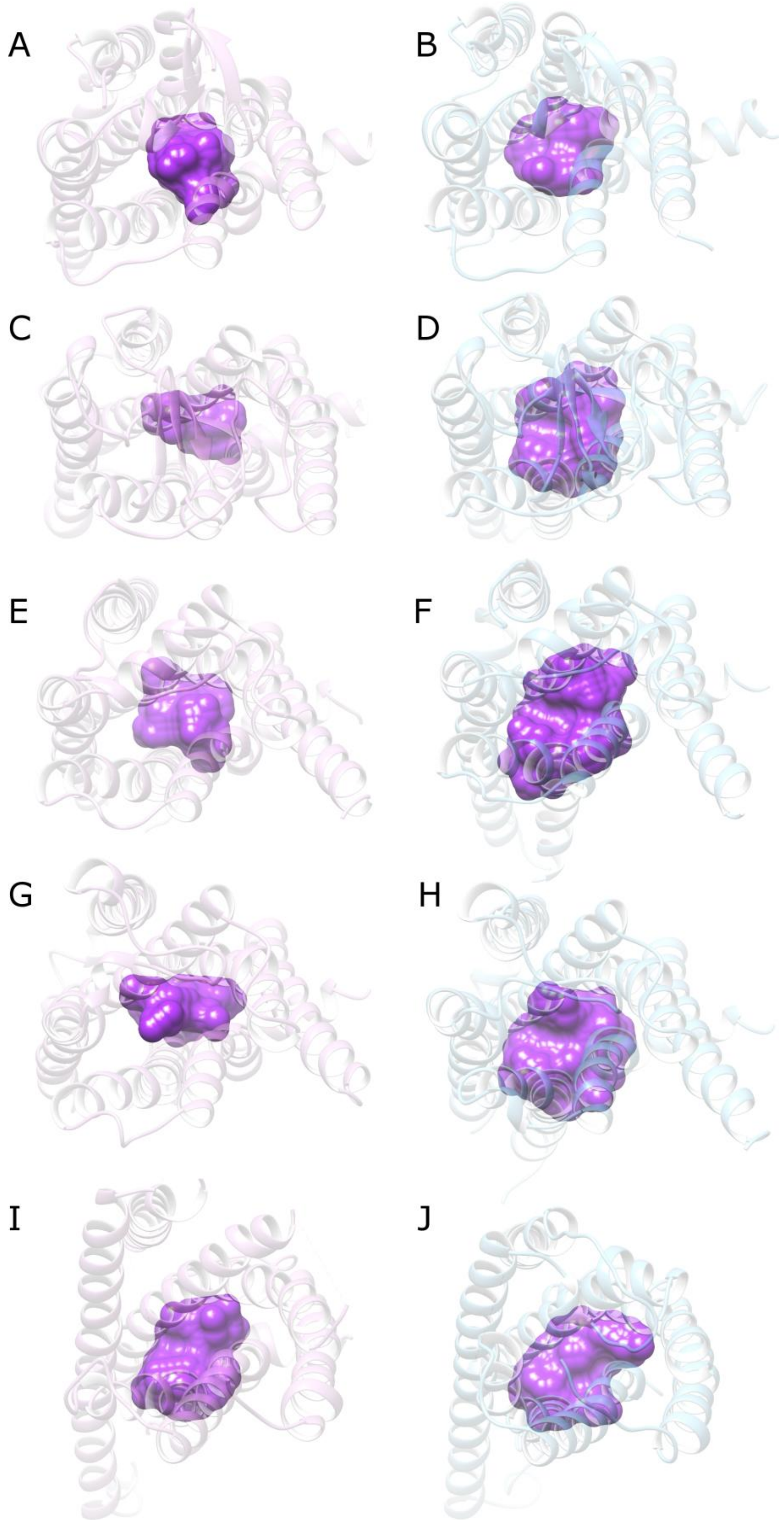

SI Fig. 5 Comparison of inactive states (light pink) and active states (light blue) of Class A GPCRs (extracellular view): (A) and (B): adenosine A2A receptor, (C) and (D): rhodopsin, (E) and (F): ß2-adrenergic receptor, (G) and (H): muscarinic acetylcholine M2 receptor, (I) and (J): P2Y purinoceptor12. Intracellular G-protein binding-site volumes (in purple) were calculated with POVME [2].

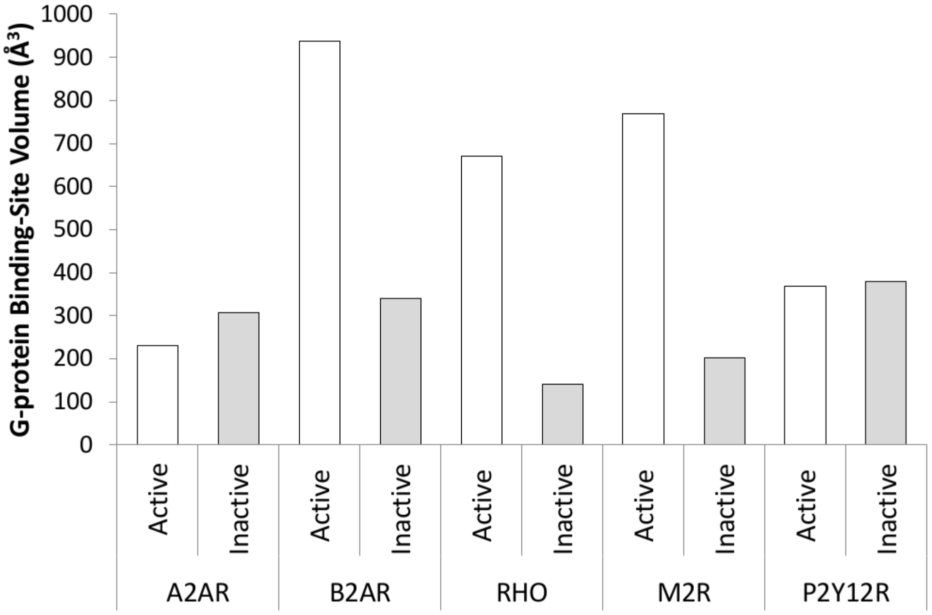

SI Fig. 6 Intracellular G-protein binding-site volumes (Å^3) in the inactive and active states of Class A GPCRs: ß2-adrenergic receptor (B2AR), rhodopsin (RHO), adenosine A2A receptor (A2AR), muscarinic acetylcholine M2 receptor (M2R), P2Y purinoceptor 12 (P2Y12R).

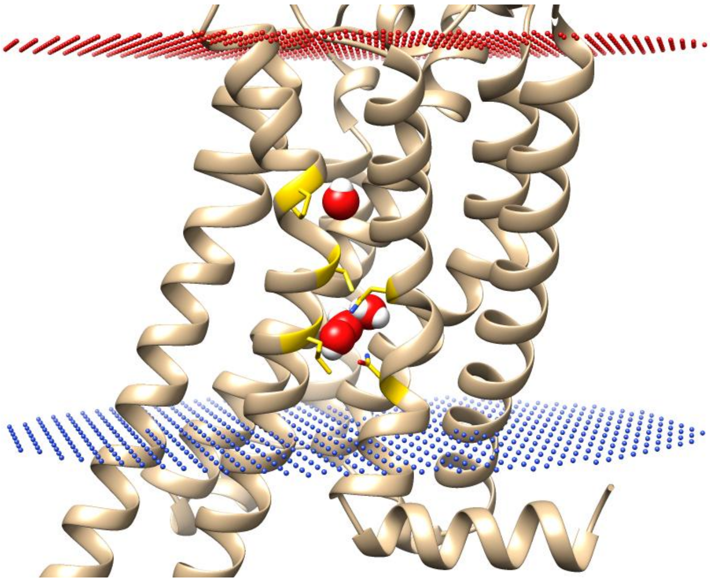

SI Fig. 7 Visualization of the water-mediated H-bonding network between TM6 and TM7 in high-resolution Adenosine A2A Receptor (-BRIL fusion, PDB id: 4EIY) structure [3] in its inactive state with residue positions L6.43, C6.47, P6.50, N7.45, N7.49 highlighted (in gold) (Ballesteros-Weinstein numbering [1]) and 4 water molecules (red/white spheres). If mutated, these residues cause an increase in constitutive activity of some Class A GPCRs (see main text). The extracellular side of the membrane is represented as red dots and the intracellular side by blue dots.

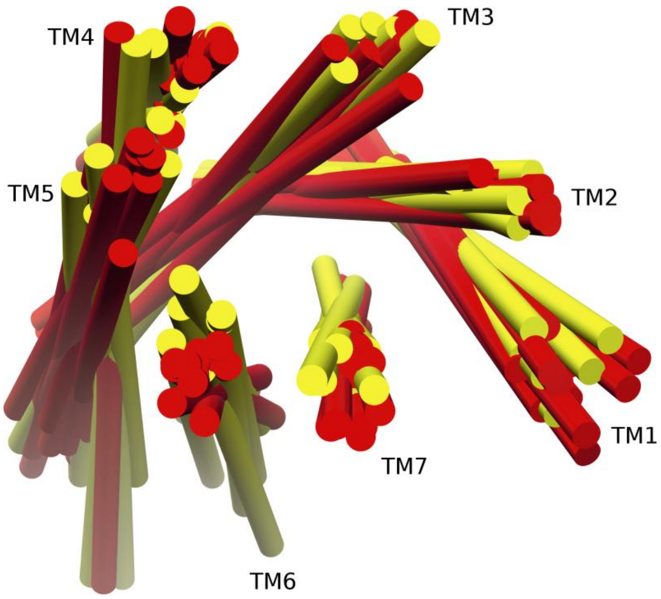

SI Fig. 8 An ensemble of 7TM conformations (extracellular view, helices represented as cylinders, calculated with CHIMERA [4]) for inactive (red, 10 structures) and active (yellow, 6 structures) class A GPCRs.

References

1. Ballesteros J, Weinstein H: **Integrated methods for the construction of three-dimensional models and computational probing of structure-function relations in G protein-coupled receptors**. *Methods in Neurosciences* 1995, **25**:366-428.
2. Durrant JD, de Oliveira CAF, McCammon JA: **POVME: An algorithm for measuring binding-pocket volumes**. *Journal of Molecular Graphics & Modelling* 2011, **29**(5):773-776.
3. Liu W, Chun E, Thompson AA, Chubukov P, Xu F, Katritch V, Han GW, Roth CB, Heitman LH, AP IJ *et al*: **Structural basis for allosteric regulation of GPCRs by sodium ions**. *Science* 2012, **337**(6091):232-236.
4. Pettersen EF, Goddard TD, Huang CC, Couch GS, Greenblatt DM, Meng EC, Ferrin TE: **UCSF chimera - A visualization system for exploratory research and analysis**. *Journal of computational chemistry* 2004, **25**(13):1605-1612.
